# Differential Extinction and the Contrasting Structure of Polar Marine Faunas

**DOI:** 10.1371/journal.pone.0015362

**Published:** 2010-12-22

**Authors:** Andrew Z. Krug, David Jablonski, Kaustuv Roy, Alan G. Beu

**Affiliations:** 1 Department of Geophysical Sciences, University of Chicago, Chicago, Illinois, United States of America; 2 Section of Ecology, Behavior and Evolution, Division of Biology, University of California San Diego, La Jolla, California, United States of America; 3 Institute of Geological and Nuclear Sciences Limited, Lower Hutt, New Zealand; Field Museum of Natural History, United States of America

## Abstract

**Background:**

The low taxonomic diversity of polar marine faunas today reflects both the failure of clades to colonize or diversify in high latitudes and regional extinctions of once-present clades. However, simple models of polar evolution are made difficult by the strikingly different faunal compositions and community structures of the two poles.

**Methodology/Principal Findings:**

A comparison of early Cenozoic Arctic and Antarctic bivalve faunas with modern ones, within the framework of a molecular phylogeny, shows that while Arctic losses were randomly distributed across the tree, Antarctic losses were significantly concentrated in more derived families, resulting in communities dominated by basal lineages. Potential mechanisms for the phylogenetic structure to Antarctic extinctions include continental isolation, changes in primary productivity leading to turnover of both predators and prey, and the effect of glaciation on shelf habitats.

**Conclusions/Significance:**

These results show that phylogenetic consequences of past extinctions can vary substantially among regions and thus shape regional faunal structures, even when due to similar drivers, here global cooling, and provide the first phylogenetic support for the “retrograde” hypothesis of Antarctic faunal evolution.

## Introduction

The latitudinal diversity gradient (LDG) is the most pervasive biodiversity pattern on Earth, with a dramatic pole-to-equator rise in morphological and taxonomic diversity in most marine and terrestrial clades [1]. Hypotheses about the origin and maintenance of the LDG generally focus on high tropical diversities, leaving the polar regions relatively neglected despite their intrinsic importance both as end-members of the LDG and as unique and vulnerable ecosystems. Low polar diversities are the net result of in-situ origination, immigration/emigration, and extinction of previously established lineages, but these dynamics remain poorly understood. The compositions of modern benthic marine communities in the two polar regions differ dramatically, with the Antarctic generally being as or more diverse than the Arctic [2], [3], [4] but ecologically more reminiscent of Paleozoic or early Mesozoic than of modern marine communities [5]. These differences preclude simple models of polar evolution and indicate divergent evolutionary histories for the two regions. A deeper knowledge of polar histories is thus desirable from a theoretical standpoint [6] and to inform models of biotic responses to future high-latitude climate change. Here we use marine bivalves, with a rich fossil record and a well-documented present-day biogeography [7], [8], as a model system to evaluate extinction patterns and their phylogenetic consequences at the two poles.

## Results

### Phylogenetic Structure to Extinction

**Figure 1:**
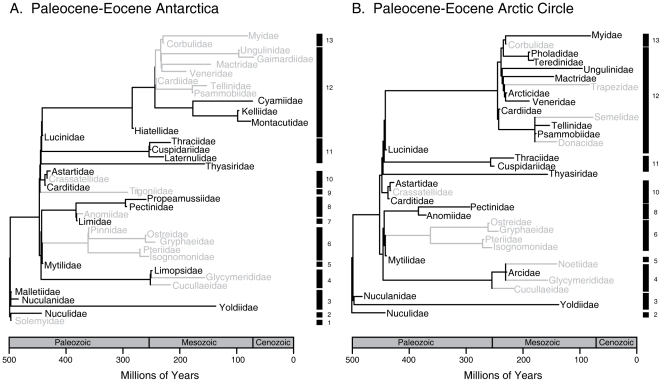
Subset of bivalve phylogenetic tree present in A. Antarctica and B. the Arctic Circle in the Paleocene and Eocene. Taxa that went locally extinct in these regions in the Modern are gray-shaded, along with the internal branches removed from the tree as a result of these absences. Internal nodes are scaled to first appearance of each family in the fossil record. Numbers and bars along the right edge demark family groupings within orders, following Bieler & Mikkelsen [44], except placement of Thyasiridae outside of (Veneroida + Lucinidae), following Taylor et al. [50]. 1. Solemyoida, 2. Nuculoida, 3. Nuculanoida, 4. Arcoida, 5. Mytiloida, 6. Pterioida, 7. Limoida, 8. Pectinoida, 9. Trigonioida, 10. Carditoida, 11. Anomalodesmata, 12. Veneroida, 13. Myoida.

In the early Cenozoic, the Arctic and Antarctic had 35 and 40 recorded bivalve families, respectively, the difference likely reflecting the poorer Arctic fossil record, with twenty-seven families shared by the two regions during the Paleocene/Eocene (Table S1). Thus the faunas began the Cenozoic with similar compositions, but since then 20 of the 40 Antarctic families, but only 12 Arctic families, have gone extinct regionally (Fig. 1a,b).

**Figure 2:**
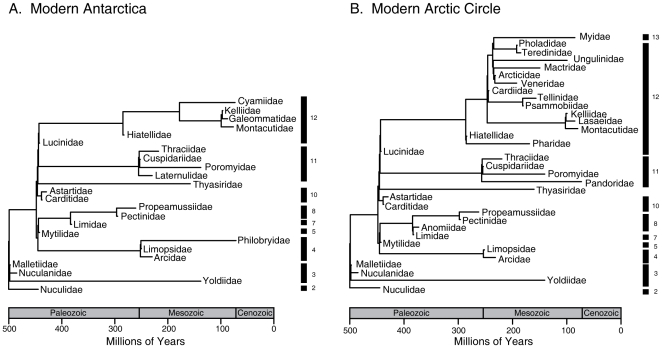
Subset of bivalve phylogenetic tree present in A. Antarctica and B. the Arctic Circle in the Modern. Note that while most of the extinctions in Antarctica are permanent, most of the evolutionary history in the Arctic tree is retained owing to the persistence/introduction of sister families of those that went extinct in this region. Family groupings within orders marked as in figure 1.

Extinction in Antarctica is strongly (D = .097) and significantly (Standardized Effect Size _MPD_  = −1.8, *p* = .03; SES_MNTD_  = −2.5, *p* = .003;) clustered phylogenetically, with extinctions concentrated in the orders Myoida, Veneroida and Pterioida (see Figure S1, Table S2 for ordinal assignments), and the family-level phylogenetic history of the Antarctic fauna was reduced by at least 27–38%, depending on the metric (see Materials and Methods). The loss of the myoids and most veneroids is particularly striking because their extinction eliminates the most derived portions of the evolutionary tree, with the remaining families largely representing more basal lineages (Fig. 2a).

The Arctic, in contrast, lost few derived families, with many veneroid and myoid families present in both the Paleocene and the Recent (Fig. 1b) or replaced by sister-families (Fig. 2b). The Pterioida, which also went extinct in the Antarctic, is the only ordinal-level loss in the Arctic (Fig. 1b). The Arctic extinctions exhibit much weaker phylogenetic clustering (D = .479; SES_MPD_  = −.14, *p* = .45; SES_MNTD_ = −1.3, *p* = .07) and the family-level tree remained topologically stable (total branch lengths, Paleocene/Eocene  = 2965.12 Myr, Modern  = 2819.91 Myr, Fig. 1b, 2b).

### The Effects of Species and Genus Richness on Family-Level Extinction Dynamics

The above results show that the differences in modern polar bivalve faunas arose via phylogenetic selectivity of Cenozoic family-level extinction in Antarctica that was absent in the Arctic. The use of families as terminal taxa simplifies an underlying distribution of species and genus richnesses within these families that might enhance, through probabilistic effects, the chances of producing phylogenetically clumped extinctions in Antarctica but not the Arctic (for example, if closely related families all had lower species richnesses in Antarctica, random species-level extinction could produce the observed phylogenetic signal). However, several lines of evidence suggest that neither species nor genus richnesses of families drove the patterns.

First, the age-frequency distributions of genera at both poles are statistically indistinguishable (Figure S2), and genus-level Lyellian percentages (the percent of genera within a time interval that survive to the Recent [9]) for both the Paleocene/Eocene and Pliocene of the Arctic and Antarctic are comparable (Table 1), indicating that turnover rates of genera were similar in the two regions throughout the Cenozoic. Second, the derived families that preferentially go extinct in Antarctica generally have lower rates of extinction at the global scale [10], meaning the global intrinsic extinction rate of the family is likely decoupled from the regional Antarctic pattern.

**Table 1:** Lyellian percentages for the Arctic and Antarctic faunas of the Pliocene and Paleocene/Eocene intervals.

Region	Time Interval	% Genera Surviving	Lower 95% CI	Upper 95% CI
Arctic	Pliocene	87	78	94.9
	Paleocene/Eocene	31	19.4	43.3
Antarctic	Pliocene	92	74.1	100
	Paleocene/Eocene	22	13.9	31.1

Data from the Paleocene/Eocene interval are limited to the genus level and come from datasets collected for this manuscript (see SI Table 1 for references). Arctic Pliocene percentages from Valentine et al. 2008 [52]. Antarctic Pliocene percentages were obtained from references listed in Beu 2009 [15]. Confidence intervals were calculated using binomial probabilities on percentage data as presented in Raup 1991 [53].

Finally, the phylogenetic signal to Antarctic extinction is unrelated to genus-level dynamics. To test this, we randomly selected genera, without replacement, from the Paleocene/Eocene faunal list and designated them as extinct. We then determined which families would have gone extinct given the random draw of genera, calculated MPD and MNTD for these families, repeated the process 10,000 times, and determined summary statistics. Lyellian percentages were used to determine the proportion of surviving genera in each region. The results are incompatible with the observed family-level extinction patterns, as family-level extinction intensity is significantly higher in the randomizations (recorded Antarctic extinction: 20 families; mean random extinction: 25±1.5 families; 95% CI calculated as 1.96*standard deviation of randomized extinction). Furthermore, although the random extinctions could produce high MPD and MNTD values, those rarely came from the extinction of derived families. The veneroid and myoid families that produce the signal in Antarctica went extinct simultaneously in only 0.3% of the runs, while the pterioid order was completely removed in only 15% of the runs (this order went completely extinct in both poles). The significant phylogenetic pattern in Antarctica is therefore unlikely to result simply from independent genus-level extinctions in this region.

Comparable randomizations could not be performed using species-level Lyellian percentages, as turnover at this taxonomic level was too high. However, the difference in family extinctions between the Arctic and Antarctic is likely unrelated to probabilistic effects of species-richness, as (a) species richness of families in the Arctic and Antarctic in the Paleocene/Eocene are correlated (Spearman's rho  = 0.6, *p* = .0003) and (b) families occurring at both poles in the Paleocene/Eocene tended to have more species in the Antarctic (Wilcoxon signed ranks test, *p* = .005), including high-diversity families such as Mactridae and Veneridae, which went extinct in Antarctica but persist in the Arctic. This contrast between the poles conceivably might arise by poor Arctic sampling, but species richness of a family in the Paleocene/Eocene is also uncorrelated with its probability of extinction in Antarctica (Kolmogorov-Smirnov test, *p* = .06, this test actually suggests an inverse relationship between Paleocene/Eocene species richness and survivorship in Antarctica), making it unlikely that stochastic effects predicated on species richness produced the strong phylogenetic signal there.

## Discussion

These results provide phylogenetic confirmation that the seemingly archaic nature of the Antarctic marine fauna reflects the loss of derived clades [5], [11], rather than long-term phylogenetic stasis with exclusion of derived clades since the late Mesozoic. In-situ diversification certainly occurred, but almost exclusively within the basal clades.

Though the timing of the extinctions at the poles is not well resolved given the sparse fossil records, climatic cooling may be a primary cause of Cenozoic polar extinctions given (a) observed correlations between modern sea surface temperature and diversity [12], [13] and (b) the Antarctic faunas of the Oligocene and Early Miocene, immediately following the onset of glaciation there [14], appear to be missing many of the derived families [15], although sampling in this time interval remains sparse. More importantly, the strong phylogenetic component to Antarctic extinctions demonstrates that shared evolutionary history can lead to shared extinction risk within regions, a pattern previously demonstrated only at global scales [10]. The differences between Arctic and Antarctic extinction patterns, particularly the importance of phylogenetic position only in Antarctica, could therefore reflect (a) differences in the adaptive landscapes (perhaps due to the magnitudes and trajectories of environmental or biotic changes) at the two poles or (b) the presence of shared traits and/or adaptations in Antarctic species that never evolved in the Arctic or *vice versa*. However, basic molluscan functional attributes (including life position, feeding strategy, fixation, and mobility [16]; Figure S3) or reproductive strategies do not appear to differ substantially among the two regions. Antarctic mollusks are dominated by non-planktotrophic developers, but several bivalve clades contain planktotrophic species there [17], some of which are ecologically dominant [18], and clades absent from the Antarctic are capable of producing non-planktotrophic species at small body sizes or high northern latitudes [19]. Thus while we cannot rule out the role of other traits that are not preserved in the fossil record (e.g. differences in metabolic rates, thermal adaptations, or larval durations), differential changes in the biotic or physical environments are more likely to have caused the difference in the phylogenetic patterns of polar extinctions.

At least four nonexclusive hypotheses have generally been framed for the evolution of Antarctic faunas, and all could have played a role in driving the differential extinction patterns:

### 1) Effect of glaciation

Continental loading of glaciers in Antarctica, beginning ∼33.5 Ma [14], [20], produced unique environmental conditions, including steep, narrow continental shelves and restricted shallow-water habitats [3], [21], [22], the preferred environment for many veneroid families [15]. Arctic sea ice formed much later (∼12–24 Ma [14], though the first ice-rafted debris occurs at ∼45 Ma [23]), and left more shallow-water shelf habitats accessible to biota [22]. Though glacial history probably accounts for some of the polar contrast in extinction, it probably cannot fully explain the strong phylogenetic clustering of extinctions in the Antarctic. The shelf environments of the Weddell and Ross Seas contain the basal infaunal families (eg. yoldiids, laternulids, thraciids), but not the more derived ones that were present in the Paleocene/Eocene, as well as families that co-occur at shelf depths with the more derived clades at lower latitudes (e.g. arcoids, a few small mytilids, and one pectinid species). Our inclusion of the Scotia Arc islands as part of the Antarctic fauna (Figure S4) also adds non-glaciated coastline to the region, but adds only Hiatellidae and Lucinidae (1 species each) to the fauna (Table S1).

### 2) Degree of geographic isolation

Antarctica began the Cenozoic in temperate climates and directly linked to South America, New Zealand and Australia [24], but became progressively isolated as these continents separated, the climate cooled, and the Antarctic Circumpolar Current was established [25], [26]. The Circumpolar Current is not impermeable [25], [26], [27], but the Antarctic is strongly isolated and accessible to temperate faunas primarily via the Scotia Arc islands. In contrast, the Arctic ocean basin was enclosed in the Paleocene and progressively opened through the Cenozoic [28]. All major coastlines and several ocean currents extend from the temperate zone to the Arctic Ocean, and temperate faunas have migrated through the Arctic into other ocean basins repeatedly in the late Cenozoic [29]. Thus the Arctic could have sustained the derived bivalve families through continual replenishment from temperate sources even in the face of regional extinctions. Any derived clades lost from Antarctica during the early Cenozoic would have been less likely to return.

### 3) Extinction of durophagous predators

Shell-crushing predators, which diversified globally on shallow shelves in the Mesozoic and Cenozoic, and have influenced the evolution of their benthic prey [30], disappeared from Antarctica [31]. In the absence of fast moving, powerful predators, the costs of skeletal defenses and relatively high metabolic rates found in derived bivalve families might be disadvantageous, particularly in cold environments where temperature adaptations are themselves costly and figure into evolutionary tradeoffs [32], and hence could have produced a phylogenetically clustered extinction. It is not yet clear that these factors are sufficient to cause regional extinctions. However, experimental tests for tradeoffs disfavoring post-Paleozoic clades at low temperatures, using species today living in cool-temperate climates similar to those in the Antarctic Eocene, could evaluate this hypothesis.

### 4) Cenozoic Changes in Primary Productivity

**Figure 3:**
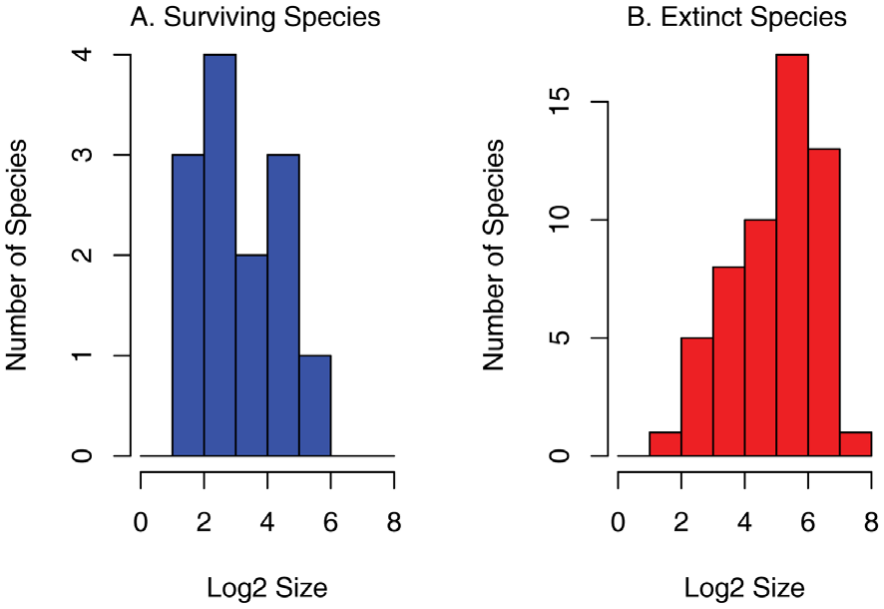
Body size distributions for species within genera that A. went locally extinct in Antarctica since the Paleocene-Eocene and B. are still present in Antarctica. Body sizes are taken as Log_2_(SQRT L * H), where L is the length and H is the height of the bivalve shell [51]. Distributions are significantly different (KS test, *p* = .003).

Shifts in primary productivity through the Cenozoic could have contributed to Antarctic extinctions. Reported rates of net primary productivity (NPP) in Antarctica today are generally low, despite a lack of nutrient limitation, when compared to other biogeographic provinces [3], [33], [34], [35]. However, estimates of Antarctic NPP are complicated by the uncertainties of satellite-based estimates of surface production in low temperature, high chlorophyll regions [36]. Recent studies have suggested that correcting for these problems can produce comparable rates of NPP at the two poles [37]. Body sizes of Eocene Antarctic bivalves, which can correlate with productivity [38] (among other variables), are indistinguishable from the modern global distribution and from Eocene Arctic body sizes specifically, but living Antarctic bivalves are significantly smaller than both (Kruskal-Wallis test, *p* = .0006; [39]). Genera that went extinct in Antarctica since the Paleocene-Eocene contained significantly larger species than those that survived (KS test, *p* = .003; Fig. 3), and families with large mean and median body sizes today were preferentially removed from Antarctica (KS test, *p* = .003). Direct measurements comparing Paleocene-Eocene and Recent productivity rates from the two poles are lacking. However, the similarities in Paleocene-Eocene body sizes and taxonomic compositions coupled with warmer climates during this time [14], [20] all suggest that Early Cenozoic productivity rates in Antarctica were comparable to those in the Arctic of the time, but dropped more rapidly as temperatures cooled and the continent became isolated, potentially resulting in a history of extinction distinct from that of the Arctic. This hypothesis will be better evaluated as robust estimates of modern polar productivity become available.

Quantifying the differential phylogenetic patterns of family loss at the two poles sharpens our understanding of the evolution of modern marine biodiversity patterns [40]. The importance of extinction in shaping global diversity patterns is increasingly well documented, but the phylogenetic legacy of past extinctions on regional faunas is often disregarded even though it can be as important as patterns of speciation in shaping modern diversity trends. Our results also demonstrate that shared evolutionary history determined extinction risk in Antarctica but not the Arctic, despite their similar Paleocene/Eocene family compositions. The predominantly basal clades that remained after the phylogenetically clumped Antarctic extinctions define the retrograde nature of communities there, with the similar diversities at the two poles underscoring the distinction between phylogenetic and taxonomic diversity, as increasingly appreciated from a conservation standpoint [41], [42].

## Materials and Methods

The origins of phylogenetic contrast between Arctic and Antarctic faunas were evaluated using fossil and living marine bivalves. Bivalves provide an excellent model system for such analyses because their LDG closely parallels the global pattern, and both modern and early Cenozoic polar faunas have been well-sampled and taxonomically standardized, providing a direct window into the composition of pre-glaciation faunas. Paleocene-Eocene faunas (65–34 Myr ago) are well-preserved in Antarctica and have received considerable study (Figure S4, Table S1). The Paleocene/Eocene has a sparser fossil record in the Arctic, but faunas from northwest Greenland and Spitsbergen were deposited above 70° N (Figure S4, Table S1). Family richnesses and compositions of these Arctic and Antarctic macrofossil assemblages are similar and span the bivalve phylogeny (Fig. 1), suggesting that differential sampling effects are minor at the family level. We focused on comparisons of Early Cenozoic and Recent assemblages, as the Miocene-Pleistocene macrofossil record in Antarctica is poor, the mid-Cenozoic Arctic Ocean was not fully marine [43], and the Pliocene-Pleistocene Arctic and Antarctic faunas were essentially modern at the family and genus level (Table 1).

The phylogenetic hypothesis used here is a composite family-level tree integrating several published molecular phylogenies [10], [44] (Figure S1). The internal branch lengths of the tree were scaled using the geologic ages of the families, so that each node is assigned the age of the oldest included family [10]. Modern and fossil assemblages were compared by estimating the total length of the branches, as both Myr and number of nodes, in the phylogenetic trees of the different regions using families as terminal taxa; this is similar to the phylogenetic diversity metric (PD) [42] used in conservation biology and biogeography [45], which uses all internal branches with species as terminal taxa. Because the extinct species are missing from our molecular tree, our branch lengths provide only a minimum estimate of true PD. However, because species richnesses in the two regions were comparable during the early Cenozoic, relative comparisons of loss of deeper evolutionary history due to extinction should be possible. Using these metrics, dramatic reductions in evolutionary history can only occur through phylogenetically non-random extinction [41], as internal branches can be recorded by multiple taxa, requiring the extinction of all of them to collapse the tree. Because modern faunas are better preserved and sampled than fossil ones, regional extinctions can be read directly from the fossil record as minimum estimates, whereas additions in modern faunas cannot be accepted at face value.

Phylogenetic clumping in extinction was evaluated using two measures of phylogenetic relatedness of taxa, mean pairwise distance (MPD) and mean nearest taxon distance (MNTD). A matrix of pairwise distances was calculated using the global bivalve phylogeny and used to determine the MPD and MNTD of extinct families in the Arctic and Antarctic. Statistical significance of the clumping was determined using the standardized effect size (SES), which compares the computed distances to those calculated by randomly swapping the tips of the regional phylogenetic trees; 10,000 randomizations were performed. Negative SES values indicate smaller distances between taxa than expected (i.e. phylogenetic clumping). Additionally, the strength of the phylogenetic signal in each polar fauna was calculated using the D statistic [46], allowing a more detailed comparison of the importance of phylogenetic patterning among regions than significance tests alone can provide. The strength of the phylogenetic signal increases as D decreases, with values of 1 indicating the trait has a phylogenetically random distribution and 0 indicating strong clumping expected if the trait evolved under a Brownian evolutionary model [46]. All calculations were done using R [47]. MPD and MNTD were calculated using the package ‘picante’ [48], and D using the package CAIC [49].

## Supporting Information

Figure S1 **Phylogenetic hypothesis of the relationships between living bivalve families.** 67 of the ∼100 living families of bivalves could be confidently placed on the tree. The tree includes all families present in the Paleocene or Eocene of the Arctic or Antarctica. Numbers and bars along the right edge demark family groupings within orders, following Bieler & Mikkelsen [44]. 1. Solemyoida, 2. Nuculoida, 3. Nuculanoida, 4. Arcoida, 5. Mytiloida, 6. Pterioida, 7. Limoida, 8. Pectinoida, 9. Trigonioida, 10. Carditoida, 11. Anomalodesmata, 12. Veneroida, 13. Myoida.
(TIF)

Figure S2 **Age‐frequency distributions for modern faunas from A. Antarctica and B. the Arctic.** Distributions are statistically indistinguishable (Kolmogorov‐Smirnov test, *p*=.6; Wilcoxon Mann‐Whitney test, *p*=.23).
(TIF)

Figure S3 **Distribution of marine bivalve families present in Antarctica in the Paleocene and Eocene among four functional categories, A. substrate affinity, B. mobility, C. feeding strategy, and D. fixation.** Families that survived to the Recent are marked in blue, those that went locally extinct in Antarctica in the Cenozoic are marked in red. The distribution of extinct versus surviving families for all 4 categories are statistically indistinguishable using a Chi‐square test (life habit: *p*=.54; mobility: *p*=.26; feeding strategy: *p*=.29; fixation: *p*=.14).
(TIF)

Figure S4 **The geographic distribution of Arctic and Arctic faunas through time.** A. Map of the world showing the geographic extent of the Arctic and Antarctic. Polar regions are denoted following Spalding et al. 2007 [54], with the exception of the subantarctic islands of New Zealand and the Indian Ocean, which now sit in polar currents but whose faunas do not interact with those of continental Antarctica. The islands of the Scotia Arc, however, are included following Zelaya 2005 [55] and Linse 2006 [56], though these islands intersect with a temperate ocean current, making their inclusion conservative. B. Map denoting continental positions in the Paleocene, redrawn from Stillwell 2003 [24]. Red dots represent localities containing bivalve fossils included in the Arctic and Antarctic, following references listed for Table S1.
(TIF)

Table S1 **Families present in the Arctic and/or Antarctic in either the Paleocene/Eocene or Modern.** Numbers refer to the references below that record the families in a time bin for a locality.
(DOC)

Table S2 **Ordinal assignments for marine bivalve families found in the Paleocene/Eocene of the Arctic and Antarctic.** Order numbers correspond to numbers in Figure S1. Note that the family Thyasiridae has not been assigned to an order, as its placement varies [44],[50]. However, this placement does not affect our results, as Thyasiridae is consistently bracketed within families that survive in both poles through the Cenozoic.
(DOC)
