# Supplementary figures and images for: Differential Extinction and the Contrasting Structure of Polar Marine Faunas
Source: PLoS One. 2010 Dec 22;5(12):e15362. doi: 10.1371/journal.pone.0015362 (PMC3008738; doi:10.1371/journal.pone.0015362)

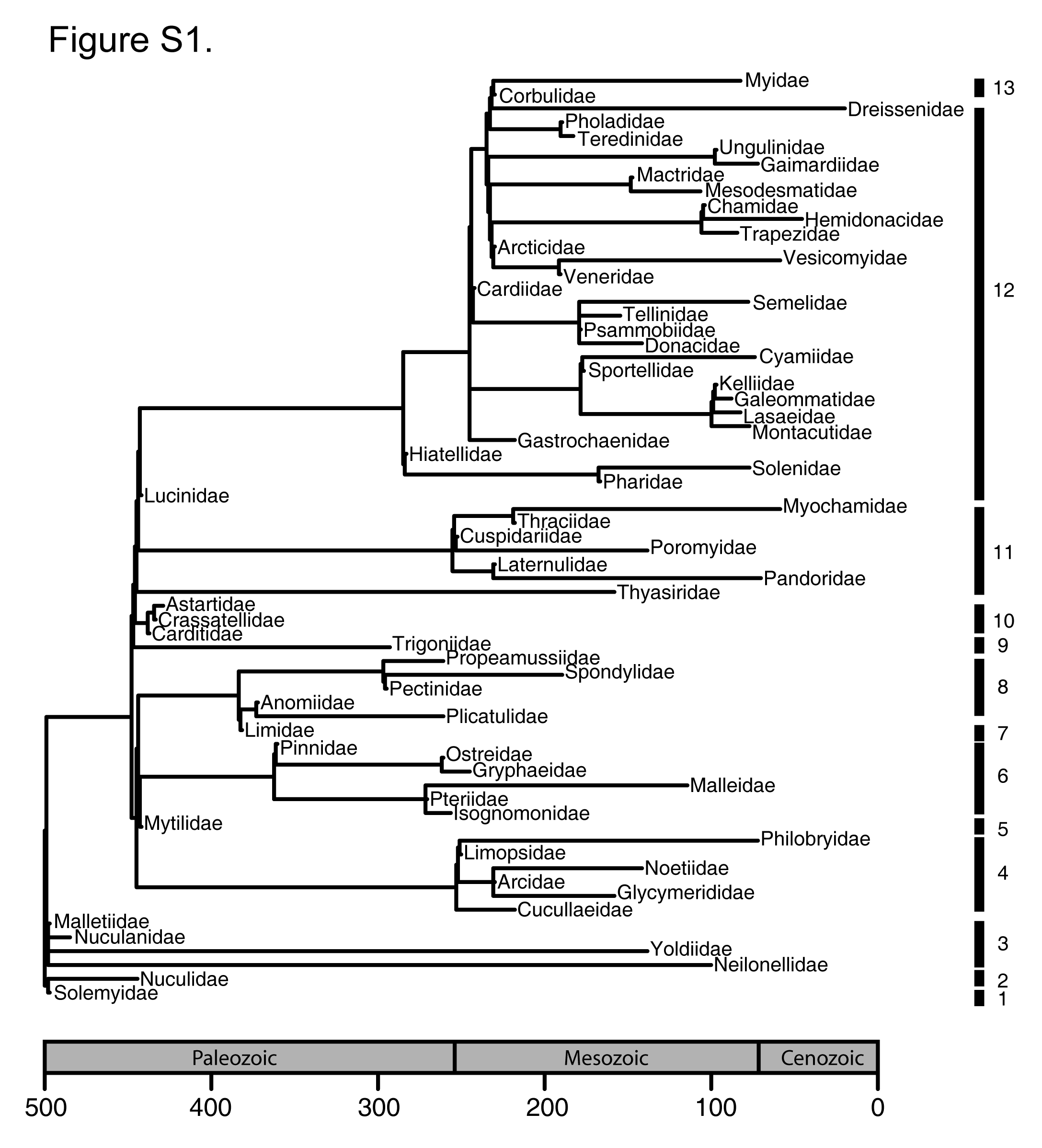

Supplement: Figure S1 — Phylogenetic hypothesis of the relationships between living bivalve families. 67 of the ∼100 living families of bivalves could be confidently placed on the tree. The tree includes all families present in the Paleocene or Eocene of the Arctic or Antarctica. Numbers and bars along the right edge demark family groupings within orders, following Bieler & Mikkelsen [44]. 1. Solemyoida, 2. Nuculoida, 3. Nuculanoida, 4. Arcoida, 5. Mytiloida, 6. Pterioida, 7. Limoida, 8. Pectinoida, 9. Trigonioida, 10. Carditoida, 11. Anomalodesmata, 12. Veneroida, 13. Myoida. (TIF) [file pone.0015362.s001.tif]

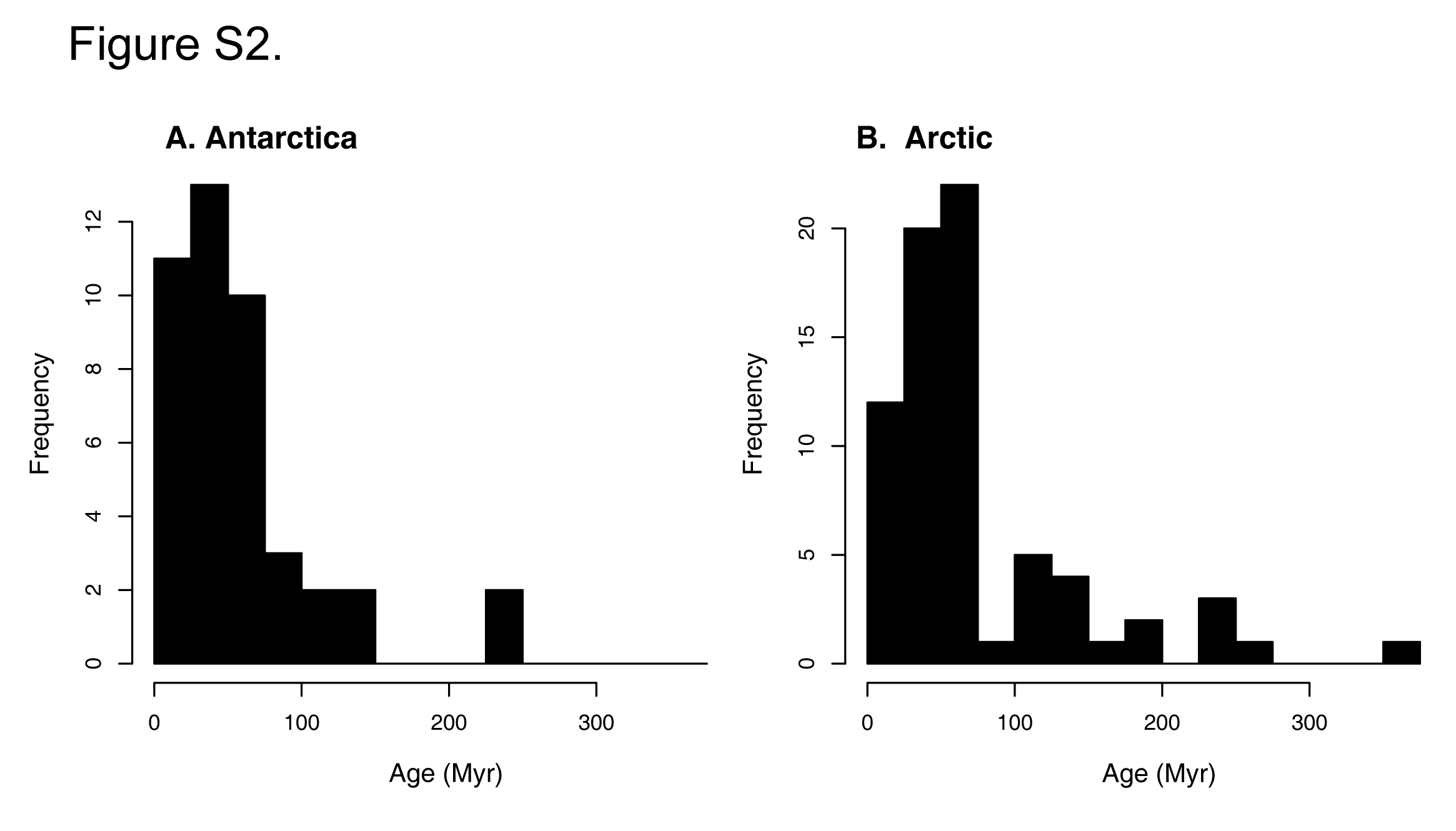

Supplement: Figure S2 — Age‐frequency distributions for modern faunas from A. Antarctica and B. the Arctic. Distributions are statistically indistinguishable (Kolmogorov‐Smirnov test, p=.6; Wilcoxon Mann‐Whitney test, p=.23). (TIF) [file pone.0015362.s002.tif]

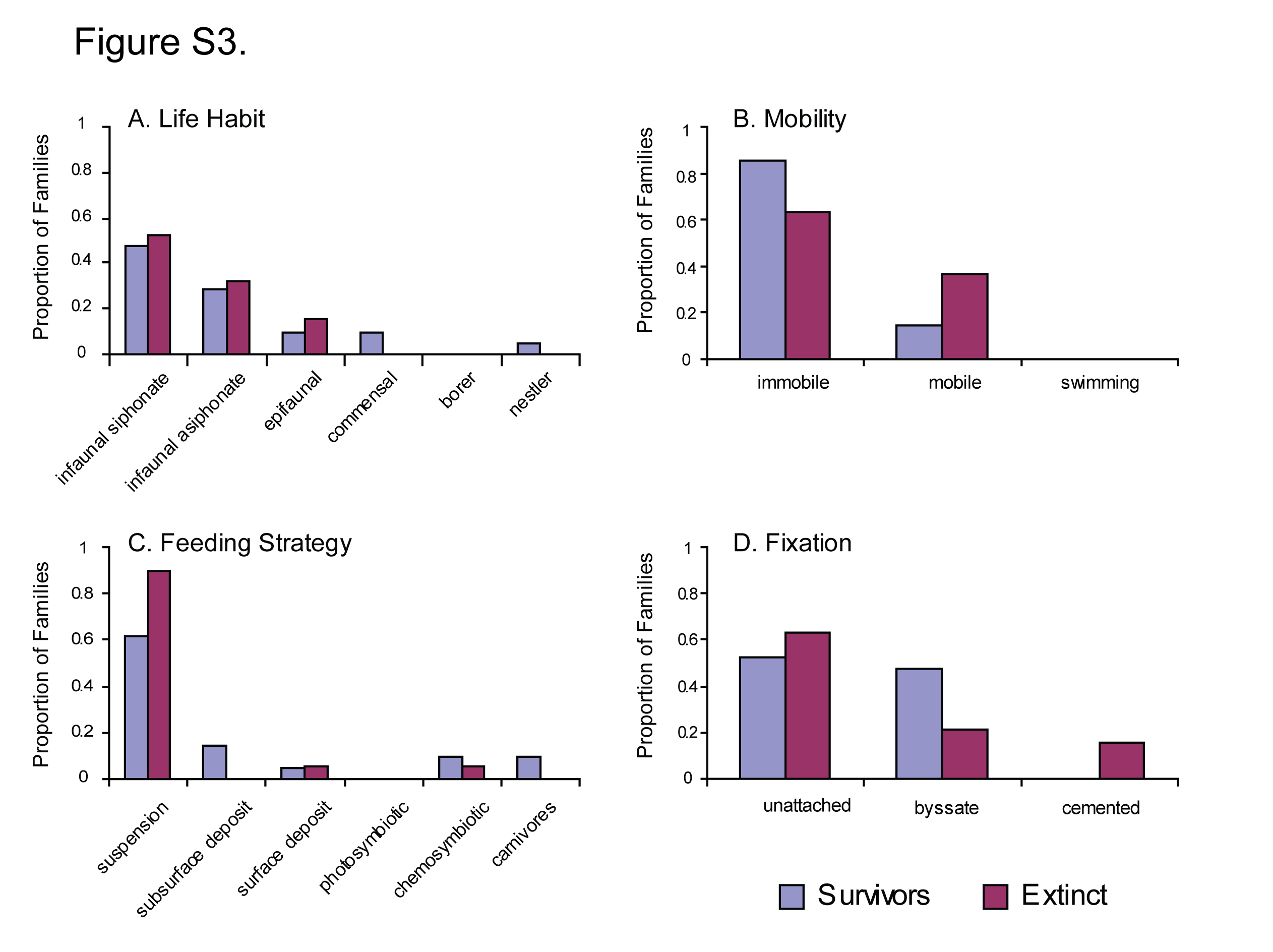

Supplement: Figure S3 — Distribution of marine bivalve families present in Antarctica in the Paleocene and Eocene among four functional categories, A. substrate affinity, B. mobility, C. feeding strategy, and D. fixation. Families that survived to the Recent are marked in blue, those that went locally extinct in Antarctica in the Cenozoic are marked in red. The distribution of extinct versus surviving families for all 4 categories are statistically indistinguishable using a Chi‐square test (life habit: p=.54; mobility: p=.26; feeding strategy: p=.29; fixation: p=.14). (TIF) [file pone.0015362.s003.tif]

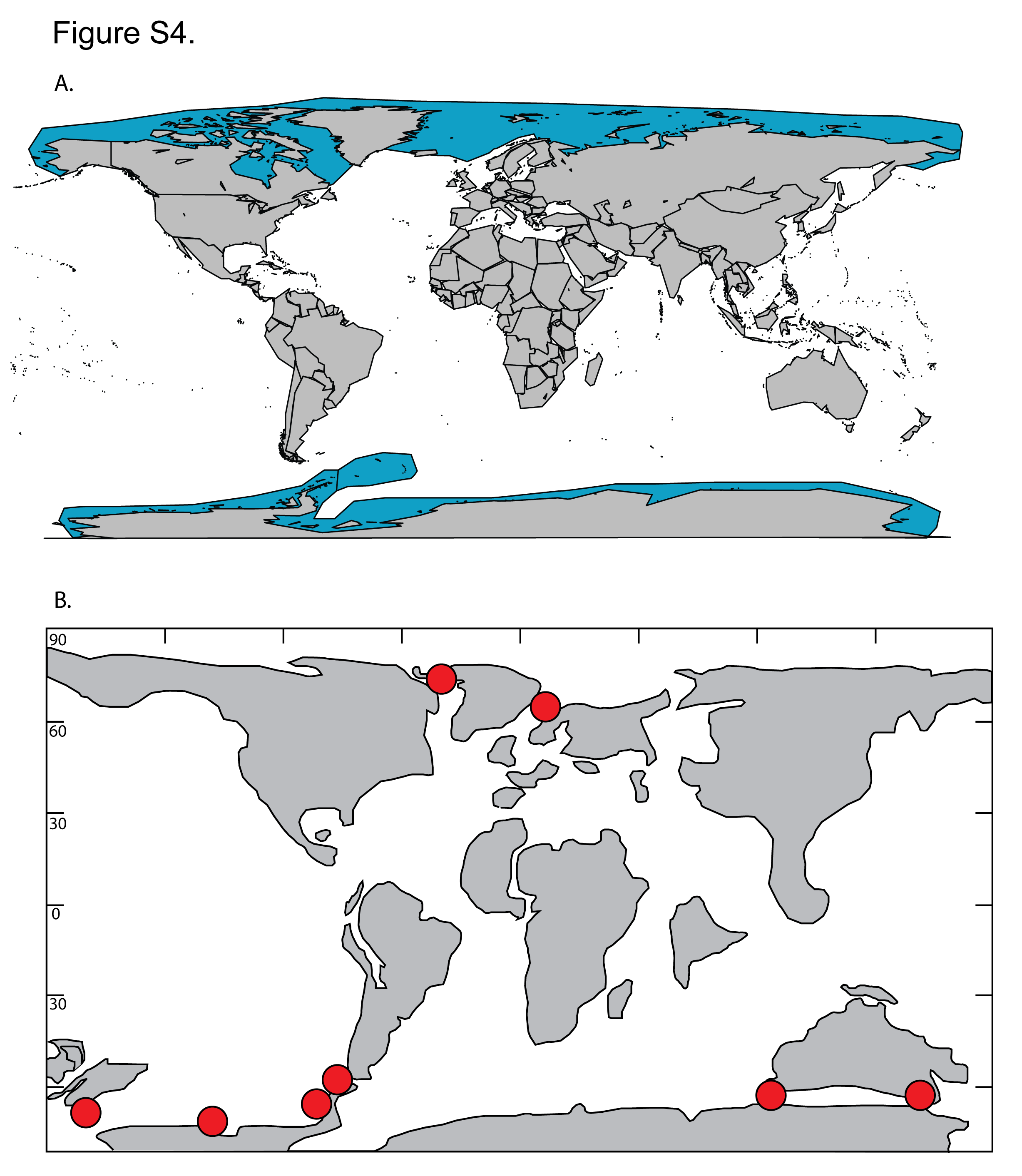

Supplement: Figure S4 — The geographic distribution of Arctic and Arctic faunas through time. A. Map of the world showing the geographic extent of the Arctic and Antarctic. Polar regions are denoted following Spalding et al. 2007 [54], with the exception of the subantarctic islands of New Zealand and the Indian Ocean, which now sit in polar currents but whose faunas do not interact with those of continental Antarctica. The islands of the Scotia Arc, however, are included following Zelaya 2005 [55] and Linse 2006 [56], though these islands intersect with a temperate ocean current, making their inclusion conservative. B. Map denoting continental positions in the Paleocene, redrawn from Stillwell 2003 [24]. Red dots represent localities containing bivalve fossils included in the Arctic and Antarctic, following references listed for Table S1. (TIF) [file pone.0015362.s004.tif]
