# Supplementary material for: Differential Extinction and the Contrasting Structure of Polar Marine Faunas
Source: PLoS One. 2010 Dec 22;5(12):e15362. doi: 10.1371/journal.pone.0015362 (PMC3008738; doi:10.1371/journal.pone.0015362)
Supplement: Table S1 — Families present in the Arctic and/or Antarctic in either the Paleocene/Eocene or Modern. Numbers refer to the references below that record the families in a time bin for a locality. (DOC) [file pone.0015362.s005.doc]

Table S1

| **Family** | **Arctic** | | **Antarctica** | |
| --- | --- | --- | --- | --- |
|  | Paleocene/Eocene | Modern | Paleocene/Eocene | Modern |
| Solemyidae | - | - | 1 | - |
| Nuculidae | 7 | 9, 10, 11, 12, 15 | 1, 2 | 3, 4 |
| Yoldiidae | 8 | 9, 10, 11 12, 15 | 1 | 3, 4, 5 |
| Nuculanidae | 8 | 9, 10, 11, 15 | 1, 2 | 4, 5 |
| Glycymerididae | 7 | - | - | - |
| Arcidae | 7, 8 | 9, 10, 11, 12 | - | 3, 5 |
| Noetiidae | 7 | - | - | - |
| Mytilidae | 7, 8 | 9, 10, 11, 12, 15 | 1, 2 | 3, 4, 5 |
| Isognomonidae | 7 | - | 2 | - |
| Pteriidae | 7 | - | 1, 2 | - |
| Malleidae | - | - | - | - |
| Gryphaeidae | 7 | - | 1, 2 | - |
| Ostreidae | 7 | - | 1 | - |
| Pinnidae | - | - | 1, 2 | - |
| Limidae | - | 10, 11, 12 | 1, 2 | 3, 4, 5 |
| Anomiidae | 7 | 12, 15 | 1, 2 | - |
| Pectinidae | 7, 8 | 10, 11, 12, 15 | 1, 2 | 3, 4, 5 |
| Carditidae | 7 | 9, 10 | 1, 2 | 3, 4, 5 |
| Crassatellidae | 7 | - | 1 | - |
| Astartidae | 7 | 10, 11, 12, 15 | 2 | 3, 4, 5 |
| Trigoniidae | - | - | 2 | - |
| Thyasiridae | 7, 8 | 9, 10, 11, 12, 15 | 1, 2 | 3, 4, 5 |
| Pandoridae | - | 10, 11, 12, 15 | - | - |
| Laternulidae | - | - | 2 | 3, 4, 5 |
| Poromyidae | - | 12 | - | 3, 4 |
| Thraciidae | 7 | 10, 11, 12, 15 | 1 | 3, 4, 5 |
| Lucinidae | 7, 8 | 13 | 1, 2 | 4 |
| Pharidae | - | 13 | - | - |
| Hiatellidae | - | 10, 11, 12, 15 | 1, 2 | 4 |
| Galeommatidae | - | - | - | 4, 5 |
| Lasaeidae | - | 11, 12 | - | 4 |
| Donacidae | 7 | - | - | - |
| Psammobiidae | 7 | 12 | 2 | - |
| Tellinidae | 7 | 9, 10, 11, 12, 15 | 1 | - |
| Semelidae | 7 | - | - | - |
| Cardiidae | 7 | 10, 11, 12, 15 | 1, 2 | - |
| Veneridae | 7, 8 | 9, 10, 11, 12 | 1, 2 | - |
| Arcticidae | 7, 8 | 15 | - | - |
| Trapezidae | 7 | - | - | - |
| Mactridae | 7 | 12 | 1, 2 | - |
| Gaimardiidae | - | - | 1 | - |
| Ungulinidae | 7 | 12 | 2 | - |
| Teredinidae | 7 | 12, 14 | - | - |
| Pholadidae | 7 | 12, 14 | - | - |
| Corbulidae | 7 | - | 1, 2 | - |
| Myidae | 7 | 9, 10, 11, 12, 15 | 1 | - |
| Cyamiidae | - | - | 1, 2 | 3, 4, 5 |
| Cuspidariidae | 7 | 9, 10, 11, 12 | 2 | 3, 4, 5 |
| Limopsidae | - | 12 | 1, 2 | 3, 4, 5 |
| Philobryidae | - | - | - | 3, 4, 5 |
| Malletiidae | - | 11 | 1, 2 | 6 |
| Cucullaeidae | 7 | - | 1, 2 | - |
| Propeamussiidae | - | 9, 12 | 2 | 3, 4 |
| Kelliidae | - | 11, 12 | 2 | 3, 5 |
| Montacutidae | - | 10, 11, 12, 15 | 1 | 3, 5 |

**Antarctica Paleocene/Eocene References**

1. Beu, A. G. Before the ice: Biogeography of Antarctic Paleogene molluscan faunas. *Palaeogeography, Palaeoclimatology, Palaeoecology* **284**, 191-226 (2009).
2. Stillwell, J. D. Patterns of biodiversity and faunal rebound following the K-T boundary extinction event in Austral Palaeocene molluscan faunas. *Palaeogeography, Palaeoclimatology, Palaeoecology* **195**, 319-356 (2003).

**Antarctica Modern References**

1. Dell, R. K. *Antarctic Mollusca with special reference to the fauna of the Ross Sea* (The Royal Society of New Zealand, Wellington, New Zealand, 1990).
2. Zelaya, D. G. The bivalves from the Scotia Arc islands: species richness and faunistic affinities. *Sci Mar* **69**, 113-122 (2005).
3. Schiaparelli, S., Lorz, A.N., Cattaneo-Vietti, R. Diversity an distribution of mollusc assemblages on the Victoria Land coast and the Balleny Islands, Ross Sea, Antarctica. *Antarctic Science* **18**, 615-631 (2006).
4. Southern Ocean Mollusc Database (SOMBASE). Available: <http://www.antarctica.ac.uk/bas_research/data/access/sombase/>. Accessed 2010, Nov 19.

**Arctic Paleocene/Eocene References***

1. Petersen, G. H. & Vedelsby, A. An illustrated catalogue of the Paleocene Bivalvia from Nuussuaq, Northwest Greenland: Their paleoenvironments and the paleoclimate. *Steenstrupia* **25**, 25-120 (2000).
2. Anderson, H. J. Geologie und fauna der Tertiaren Ablagerungen Zentral-Spitsbergens. *Norsk Polarinstitutt Skrifter* **153**, 5-120 (1970).

**Arctic Modern References**

1. Golikov, A.I. Fauna of the East Siberian Sea Part III. *Explorations of the Fauna of the Seas* **49**, 1-182 (1994).
2. Lubinsky, I. Marine Bivalve Molluscs of the Canadian Central and Eastern Arctic: Faunal composition and zoogeography. *Canadian Bulletin of Fisheries and Aquatic Sciences Bulletin* **207**, 1-111 (1980).
3. Richling, I. Arktische Bivalvia – eine taxonomische Bearbeitung auf Grundlage des materials der expeditionen Transdrift 1 und ARK IX/4 (1993) in das Laptevmeer. *Schriften zur Malakozoologie* **15**, 1-93 (2000).
4. Ockelmann, W. K. The zoology of East Greenland: Marine Lamellibranchiata. Medd. Grønland **122**, 5-256 (1958)
5. Coan, E.V., Scott, P.V., Bernard, F.R. *Bivalve Seashells of Western North America*. (Santa Barbara Museum of Natural History, 2000).
6. Madsen, F.J. in *The Zoology of Iceland* (ed. Frioriksson, A. et al.) 631-685 (Ejnar Munksgaard, Copenhagen and Reykjavik, 1949).
7. Naumov, A.D., Scarlato, O.A. & Fedyakov, V.V. Mollusks of the White Sea. *Bivalvia Opredeliteli Faune SSSR* **151**, 205-257 (1987).

* The age of the molluscan fauna of the Ocean Point Beds [57] of the Prince Creek Formation, northern Alaska, is controversial. Here we follow biostratigraphic arguments that these deposits are latest Cretaceous [58,59] rather than early Cenozoic [57,60]. Treating that fauna as early Cenozoic has a negligible effect on our results, as it would add one basal family (Malletiidae) and one more derived family (Hiatellidae) to the Arctic fauna, with effects statistically canceling out.
