# Supplementary material for: Differential Extinction and the Contrasting Structure of Polar Marine Faunas
Source: PLoS One. 2010 Dec 22;5(12):e15362. doi: 10.1371/journal.pone.0015362 (PMC3008738; doi:10.1371/journal.pone.0015362)
Supplement: Table S2 — Ordinal assignments for marine bivalve families found in the Paleocene/Eocene of the Arctic and Antarctic. Order numbers correspond to numbers in Figure S1. Note that the family Thyasiridae has not been assigned to an order, as its placement varies [44],[50]. However, this placement does not affect our results, as Thyasiridae is consistently bracketed within families that survive in both poles through the Cenozoic. (DOC) [file pone.0015362.s006.doc]

Table S2

| **Order #** | **Order** | **Included Families** |
| --- | --- | --- |
| 13 | Myoida | Corbulidae, Myidae |
| 12 | Veneroida | Lucinidae, Hiatellidae, Montacutidae, Kelliidae, Cyamiidae, Donacidae, Psammobiidae, Tellinidae, Semelidae, Cardiidae, Veneridae, Arcticidae, Trapezidae, Mactridae, Gaimardiidae, Ungulinidae, Teredinidae, Pholadidae |
| 11 | Anomalodesmata | Thyasiridae, Pandoridae, Laternulidae, Poromyidae, Cuspidariidae, Thraciidae |
| 10 | Carditoida | Carditidae, Crassatellidae, Astartidae |
| 9 | Trigonioida | Trigoniidae |
| 8 | Pectinoida | Anomiidae, Pectinidae, Propeamussiidae |
| 7 | Limoida | Limidae |
| 6 | Pterioida | Isognomonidae, Pteriidae, Gryphaeidae, Ostreidae, Pinnidae, |
| 5 | Mytiloida | Mytilidae |
| 4 | Arcoida | Cucullaeidae, Glycymerididae, Arcidae, Noetiidae, Limopsidae, Philobryidae |
| 3 | Nuculanoida | Yoldiidae, Nuculanidae, Malletiidae |
| 2 | Nuculoida | Nuculidae |
| 1 | Solemyoida | Solemyidae |
